# Study of LBHD1 Expression with Invasion and Migration of Bladder Cancer

**DOI:** 10.1515/biol-2019-0049

**Published:** 2019-11-15

**Authors:** Chunhui Dong, Yihui Liu, Guiping Yu, Xu Li, Ling Chen

**Affiliations:** 1 Department of Oncology, The First Affiliated Hospital of Xi’an Jiaotong University, 277 West Yanta Road, Xi’an, Shaanxi, 710061, P.R. China; 2 Department of Oncology, Ninth Hospital of Xi’an, Xi’an, 710054, P.R. China; 3 Center for Clinical Molecular Biology, The First Affiliated Hospital of Xi’an Jiaotong University, Xi’an, 710061, P.R. China; 4 Cancer Center, People’s Hospital of Ningxia Hui Autonomous Region, Yinchuan, 75000, P.R. China

**Keywords:** LBHD1, bladder cancer, migration and invasion, cell proliferation, siRNA, plasmids

## Abstract

LBHD1 (C11ORF48) is one of the ten potential tumor antigens identified by immunoscreening the urinary bladder cancer cDNA library in our previous study. We suspect that its expression is associated with human bladder cancer. However, the exact correlation remains unclear. To address the potential functional relationship between LBHD1 and bladder cancer, we examined the LBHD1 expression at the mRNA and protein level in 5 different bladder cancer cell lines: J82, T24, 253J, 5637, and BLZ-211. LBHD1 high and low expressing cells were used to investigate the migration, invasion, and proliferation of bladder cancer cells following transfection of LBHD1 with siRNA and plasmids, respectively. Our experiment showed that the degree of gene expression was positively related to the migration and invasion of the cancer cells while it had little effect on cell proliferation. Knocking down LBHD1 expression with LBHD1 siRNA significantly attenuated cell migration and invasion in cultured bladder cancer cells, and overexpressing LBHD1 with LBHD1 cDNA plasmids exacerbated cell migration and invasion. Nevertheless, a difference in cell proliferation after transfection of LBHD1 siRNA and LBHD1 cDNA plasmids was not found. Our findings suggest that LBHD1 might play a role in cell migration and invasion.

## 1 Introduction

Bladder cancer is one of the most common malignancies worldwide. 549,000 patients are estimated to be diagnosed with bladder cancer annually, and 200,000 die from the disease [1]. It was reported that there would be 81,190 new cases of bladder cancer and 17,240 deaths in the United States in 2018 [2]. Approximately 75% of newly diagnosed bladder cancer cases are non-muscular invasive tumors [3]. Recurrence and progression (22%) are the two major therapeutic obstacles for life-long surveillance.

The current standard examination procedure is to perform a cystoscopy and evaluate urine cytology every 3 to 4 months in the first two years, twice per year for the third to fourth years, and yearly thereafter [4]. Thus, bladder cancer becomes one of the most expensive cancers, from diagnosis to death [5, 6]. The financial burden [7], invasiveness of cystoscopy [8], as well as low sensitivity of cytology in low-grade disease [9], have led to extensive efforts to develop noninvasive biomarkers for bladder cancer. Numerous molecular assays for the diagnosis of urothelial cancer have been developed, and their clinical applications have been investigated. Commercially available bladder tumor markers include Cytology, Hematuria detection, BTA stat, BTA TRAK, NMP22, BLCA-4, Survivin, UBC, CYFRA 21-1, DD23, uCyt+, and UroVysion [10]. Although it has been shown that superior sensitivity can be achieved by using these assays compared to urine cytology, they still have many flaws, such as poor sensitivity for low-grade disease, dependence on the expertise of a pathologist, low specificity among patients with benign urologic conditions, susceptibility of results to benign urologic conditions found in both cancerous and normal regions of the bladder, expensiveness, a lack of standardization of criteria for a positive test result, and lack of specially trained personnel [11]. Therefore, none of them are included in the current clinical guidelines.

Ten immunogenic proteins including LBHD1 (C11ORF48) were identified in our previous study by immunoscreening the bladder cancer cDNA library with ten monoclonal antibodies [12]. LBHD1 is highly conserved among mammalian common ancestors, of which 55 species including chimpanzee, dog, and mouse harbor genes homologous to the human gene LBHD1. It is located on chromosome 11q12.3, which encodes two protein isoforms by alternative splicing: 31565 Da isoform 1 with 289 amino acids and 28759 Da isoform 2 with 263 amino acids. Isoform 1 is concsidered the ‘canonical’ sequence (http://www.uniprot.org/uniprot/Q9BQE6#Q9BQE6-2) Secondary structure predictions of the ‘canonical’ sequence find that alpha helices and beta folding account for 9.51% and 11.41% of the protein structure, respectively. Special structure predictions show that the protein may contain a spiral-corner-spiral structure domain, no transmembrane region, no signal peptide, and a mitochondrial targeting peptide.

There are few reports on the function of LBHD1 gene currently. Detected by Affinity Capture-MS assay, LBHD1 performs as a ubiquitin proteasome and interacts with ubiquitin C while in its native state. Protein ubiquitylation contributes to the regulation of various cellular processes including the cell cycle, protein degradation, protein transport, receptor internalization, RNA binding, signal transduction, translation, repair of DNA damage, metabolism, and ubiquitin proteasome function [13]. Whether LBHD1 acts through these cellular processes via ubiquitin C is unknown. According to ribosome profiling analysis, the LBHD1 locus contains the same genomic sequence simultaneously translated into two alternative transcripts in different frames [14]. As UniProt shows, there are two isoforms of the protein encoded by LBHD1, but what the functions of these two isoforms are and whether there is a functional difference between the structures is unknown. Some characteristics of patients with an LBHD1 gene partial deletion are intellectual disability and abnormality of the foot and the hand, while patients with a partial duplication of the LBHD1 gene show abnormal fear/anxiety-related behavior, asthma, delayed fine motor development, delayed speech and language development, epicanthus, global developmental delay, hypertelorism, long philtrum, microtia, and thin upper lip vermilion (https://decipher.sanger.ac.uk/search?q=LBHD1#consented-patients/results)

Whether LBHD1 is related to the occurrence and prognosis of bladder cancer has not been reported. In order to explore the potential functional relationship between LBHD1 and bladder cancer, the expression of LBHD1 in 5 different bladder cancer cell lines at both the mRNA and protein level was analyzed. Moreover, LBHD1 high and low expressing cells were used to study the migration, invasion, and proliferation of bladder cancer cells following transfection of the LBHD1 gene through siRNA or plasmids.

## 2 Materials and Methods

### 2.1 Cell lines and cultures

Human bladder cancer cell lines 253J, T24 and J82 were kindly provided by Dr. Leland W.K. Chung (Cedars-Sinai Medical Center, Los Angeles, California, USA). BLZ-211 as well as 5637 cells were obtained from the Translational Medicine Center, First Affiliated Hospital, School of Medicine, Xi’an Jiao tong University. The established bladder cancer cell line BLZ-211 was described previously [12, 15, 16]. Of note, all of the bladder cancer cell lines used in this study except the 253J cell line are invasive. These cells were grown in RPMI-1640 (Gibco, Gaithersburg, MD, USA) with 10% fetal bovine serum (Bio Basic, Ontario, Canada). The cultures were maintained at 37 °C and under a humidified 5% CO_2_ atmosphere.

### 2.2 Quantitative real-time polymerase chain reaction

Total RNA was extracted from bladder cancer cell lines using RNA fast 200 reagent (Fastagen, shanghai, China). Then, 1 μg total RNA from each sample was reverse transcribed to single-stranded cDNA with RNA to cDNA PrimeScript™ RT Master Mix Premix (Perfect Real-Time; Takara, Dalian, China). Quantitative real-time PCR was carried out using a SYBR Premix Ex Taq™ II (Takara) on a CFX96 real-time PCR system (Bio-Rad Laboratories, Hercules, CA, USA). Each cycle included denaturation at 95 °C for 30 sec, annealing at 56 °C for 30 sec, and polymerization at 72 °C for 20 sec. The sequences of the primers used for PCR were as follows: LBHD1 (forward, 5’- TCCCATCTGCCGTCTATTGT -3’ and reverse, 5’- CCTGGCTCTTCACTTTGGTC -3’) and GAPDH (forward, 5’-ACCACAGT CCATGCCATCAC-3’ and reverse, 5’-TCCACCACCCTGTTGCTGTA -3’). The relative expression of LBHD1 was calculated by the 2^-ΔΔCt^ method. Data were presented as the relative quantity of target mRNA normalized to the expression of GAPDH mRNA and relative to a calibrator sample. Each sample was analyzed in triplicate.

### 2.3 Western blotting

Bladder cancer cells were washed with phosphate-buffered saline and lysed in RIPA buffer (cwbiotech) containing protease inhibitor. The protein concentration was quantified using the BCA Protein Assay Kit (cwbiotech). For each sample, an amount of 20 μg total protein was separated by 12% sodium dodecylsulfate-polyacrylamide gel electrophoresis and then transferred to nitrocellulose membranes for 1 h and a half at 320 mA in transfer buffer. After blocking with 5% degreased milk at room temperature for 1 h, the membranes were incubated overnight at 4 °C with rabbit polyclonal anti-human LBHD1 (1:1000, Abcam) and mouse monoclonal anti-GAPDH (1: 2,000, cwbiotech). After washing, the membranes were incubated with the corresponding secondary antibodies conjugated with horseradish peroxidase for 1 h at room temperature. Immunoreactive bands were incubated with ECL western blotting detection reagents (Amersham Biosciences) then visualized using a western blotting detection system (ECL Plus; Amersham Pharmacia Biotech, Little Chalfont, UK). The film was scanned, and the intensity of the bands was quantified by densitometry (Image J 1.47 software) and normalized to the corresponding GAPDH bands. All of the Western immunoblots were performed at least three times.

### 2.4 siRNA knockdown and overexpression of LBHD1

Three individual siRNAs specific for LBHD1 (si-LB-390, sense, 5’-GCCGUCUAUU GUGGUGGAATT-3’ and antisense, 5’-UUCCACCACAAUAGACGGCTT-3’, si-LB-403, sense, 5’-GGAAUCCA GUGAGGUGAAUTT-3’ and antisense, 5’-AUUCACCUCACUGGAUUCCTT-3’, si-LB-688, sense, 5’-CUCCCGUGUUUGUAGAAGUTT-3’ and antisense, 5’-ACUUCU ACAAACACGGGAGTT-3’) and negative control siRNA (sense, 5’-UUCUCCG AACGUGUCACGUTT-3’ and antisense, 5’-ACGUGACACGUUCGGA GAATT-3’) were purchased from Gene-pharm (Shanghai, China). BLZ-211 cells were seeded into 6-well plates (Corning, Inc., Corning, NY, USA) at a density of 1×10^6^ cells/well and incubated at 37 °C for 24 h. 0.8 μg of the specific siRNA targeting LBHD1 or control siRNA (both from Gene-pharm, Shanghai, China) was added to 200 μl of serum-free RPMI-1640 and mixed evenly, while 20 μl of X-tremeGENE siRNA Transfection Reagent (Roche Diagnostics, Indianapolis, IN, USA) was added to another 200 μl of serum-free RPMI-1640, gently mixed evenly and placed at room temperature for 5 min. The siRNA and X-tremeGENE siRNA Transfection Reagent solutions were mixed evenly and placed at room temperature for 20 min. Then the mixture was transferred to 6-well plates, mixed evenly, and gently incubated at 37 °C. Following a 12 h transfection, the cell culture solution was changed, and 2 ml of medium was added to each well. After 24-72 h of transfection, the cells were harvested for subsequent experiments, and non-transfected cells served as blank controls. Transfection efficiency was verified using RT-PCR and western blot assay.

The human pc-LB expression vectors and empty vectors (GV230) were obtained from Shanghai Genechem Co, Ltd. (Shanghai, China). 253J cells were seeded into 6-well plates (Corning, Inc, Corning, NY, USA) at a density of 1.5×10^6^ cells/well and incubated at 37 °C for 24 h. Either 4 μg of pc-LB expression vectors or empty vectors was added to 200 μl of serum-free RPMI-1640 and mixed evenly, while 12 μl of X-tremeGENE HP DNA Transfection Reagent (Roche Diagnostics, Indianapolis, IN, USA) was added to another 200 μl of serum-free RPMI-1640 and gently mixed evenly. The Plasmids and X-tremeGENE HP DNA Transfection Reagent solutions were mixed evenly and placed at room temperature for 30 min. Then the mixture was transferred to 6-well plates, mixed evenly and gently, and incubated at 37 °C. Following a 12 h transfection, the cell culture solution was changed, and 2 ml medium was added to each well. After 24-72 h of transfection, the cells were harvested for subsequent experiments, and non-transfected cells served as blank controls. Transfection efficiency was verified using RT-PCR and western blot assay.

### 2.5 Migration and invasion assays

BLZ-211 cells LBHD1 transfected by siRNA and negative control siRNA (5×10^5^ in 100 μl serum-free medium) as well as 253J cells transfected by pc-LB plasmids and control plasmids (1×10^6^ in 100 μl serum-free medium) were seeded in the upper chamber for 12 h. The lower chamber was filled with 500 μl of RPMI-1640 medium containing 20% fetal bovine serum for 24-48 h. Cells on the upper membrane surface were wiped off using a cotton swab. Migrated cells attached to the lower membrane surface were fixed with methanol, stained with Giemsa, and counted in ten random fields (original magnification, ×200). The migration assay was conducted in a similar fashion without coating with Matrigel. Cells in 4 or 6 random fields in each well were photographed and counted under a microscope at × 200 magnification.

### 2.6 Cell viability assay

BLZ-211 and 253J cells were transfected with LBHD1 siRNA and pc-LB. Until the third day, CCK8 (7Sea Pharmatech Co. Ltd) was added to each well, and incubation was carried out at 37 °C for 4 h. Then the medium was removed, and CCK8 was added into each well. Absorbance was measured at 450 nm.

### 2.7 Statistical analysis

All of the experiments were performed at least in triplicate. Data were presented as mean ± SEM and analyzed using the SPSS 19.0 and Graphpad Prism 5. Statistical analyses were carried out using a two-tailed unpaired Student’s t-test. P<0.05 was considered a statistically significant difference.

## 3 Results

### 3.1 Expression of LBHD1 in urinary bladder cancer cells at the mRNA and protein level

**Figure 1:**
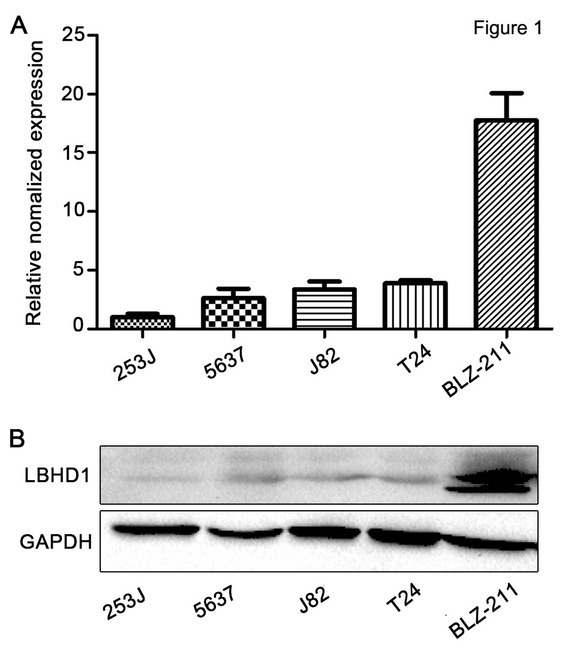
**Expression of LBHD1 in bladder cancer cells**. (A) Quantitative RT-PCR determination and (B) Western blot determination of LBHD1 expression in immortalized human bladder cancer 253J cells and bladder cancer 5637, J82, T24, and BLZ-211 cells. Transcription levels were normalized to GAPDH expression. GAPDH was used as the loading control.

The expression of LBHD1 at the mRNA and protein level was detected by RT-PCR and western blot in five bladder cancer cell lines. At the protein and mRNA level, LBHD1 was found to be highly expressed in BLZ-211 cells, whereas 253J cells exhibited the lowest expression of LBHD1 (Fig. 1). Therefore, we used BLZ-211 and 253J cells in our subsequent studies.

### 3.2 Knockdown of LBHD1 expression significantly repressed migration and invasion in bladder cancer cells

**Figure 2:**
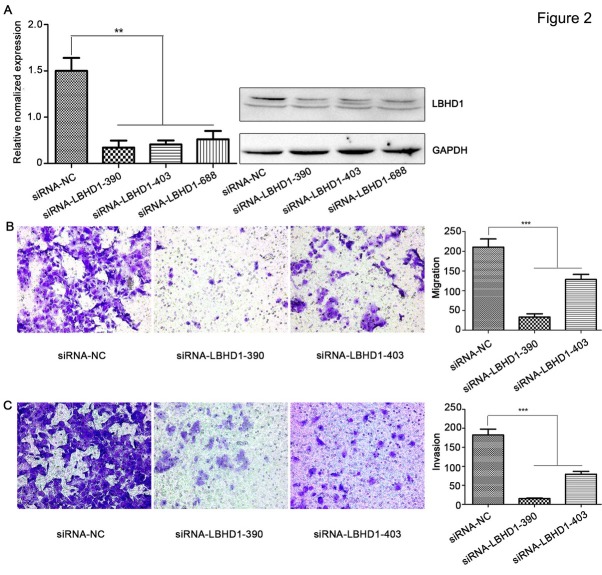
**LBHD1 knockdown inhibited the migration and invasion of bladder cancer cell line BLZ-211.** (A) Quantitative RT-PCR determination and Western blot determination of the effect of LBHD1 siRNAs (including siRNA NC, siRNA390, siRNA403, siRNA688) in bladder cancer BLZ-211 cells (^**^P<0.01). Transcription levels were normalized to GAPDH expression. (B) Cell migration assay determination of bladder cancer BLZ-211 cells following LBHD1 downregulation (^***^P<0.001). BLZ-211 cells transfected with siRNA NC, siRNA390, and siRNA403. (C) Cell invasion assay determination of bladder cancer BLZ-211 cells following LBHD1 downregulation (^***^P<0.001). BLZ-211 cells transfected with siRNA NC, siRNA390 and siRNA403.

The expression of LBHD1 was highest in BLZ-211 cells at both the mRNA (Fig. 1A) and protein level (Fig. 1B) Thus, we chose the BLZ-211 cell line for siRNA transfection. LBHD1 siRNA transfection reduced the expression of LBHD1 in BLZ-211 cells at the mRNA (Fig. 2A) and protein (Fig. 2B) level compared with negative control siRNA transfection. Knockdown of LBHD1 with siRNA decreased 67-75% of cell migration and invasion in BLZ-211 bladder cancer cells (Fig. 2B and Fig. 2C) These results suggested that LBHD1 might be involved in the migration and invasion of BLZ-211 cells and that LBHD1 siRNA negatively affected these behaviors.

### 3.3 Elevation of LBHD1 expression promoted migration and invasion of bladder cancer cells

**Figure 3:**
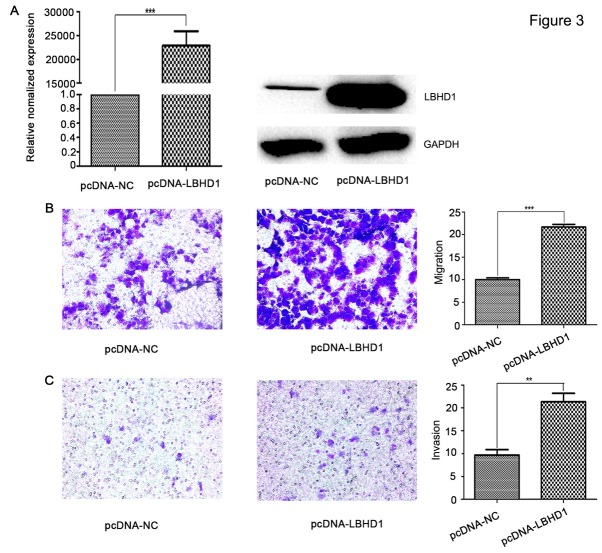
**LBHD1 overexpression enhanced the migration and invasion of bladder cancer cell line 253J.** (A) Quantitative RT-PCR determination and Western blot determination of the effect of LBHD1 expressing plasmids in bladder cancer 253J cells ( ^***^P<0.001). Transcription levels were normalized to GAPDH expression. (B) Cell migration assay determination of bladder cancer 253J cells following LBHD1 overexpression (^***^P<0.001). 253J cells transfected with NC plasmid and LBHD1 plasmid. (C) Cell invasion assay determination of bladder cancer 253J cells following LBHD1 overexpression (^**^P<0.01). 253J cells transfected with NC plasmid and LBHD1 plasmid.

Compared with BLZ-211, T24, J82 and 5637 cells, the expression of LBHD1 in 253J cells was the lowest (Fig. 1). Thus, 253J cells was selected for plasmid transfection. Total RNA was isolated from cells to quantify the expression of LBHD1 using PCR. As shown in Figure 3A the expression level of LBHD1 was high when transfected with plasmid. Control cells were transfected with vacant plasmid. Overexpression of LBHD1 increased the protein expression of LBHD1 at least 500-fold (Fig. 3A) In contrast to the siRNA knockdown of LBHD1 in BLZ-211 cells that induced cell migration and invasion repression, overexpression of LBHD1 increased cell migration by 200-319% and increased invasion in 253J human bladder cancer cells (Fig. 3B and 3C)

### 3.4 Knockdown/overexpression of LBHD1 showed no effect on cell proliferation of bladder cancer cells

**Figure 4:**
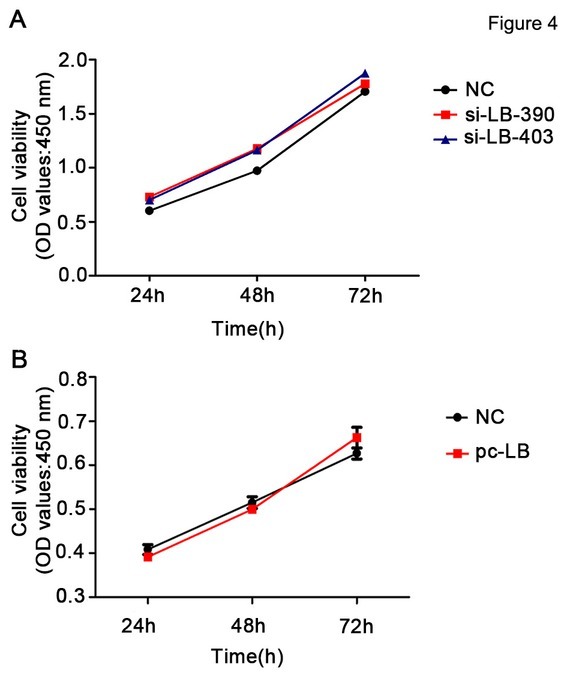
**LBHD1 knockdown or overexpression showed no statically significant effect on the proliferation of bladder cancer cell lines.** (A) CCK8 assay determination of bladder cancer BLZ-211 cell proliferation following LBHD1 downregulation regulation. (B) CCK8 assay determination of bladder cancer 253J cell proliferation following LBHD1 overexpression.

CCK8 assay detection showed that, compared with the control siRNA, LBHD1 siRNA knockdown had no significant effect on BLZ-211 cell proliferation (Fig. 4A) Effects of LBHD1 overexpression on cell proliferation were examined by comparing cell proliferation with that of cells that received the control plasmid after transient transfection of pc-LB. Consistent with the siRNA knockdown of LBHD1 in BLZ-211 cells, overexpression of LBHD1 in 253J cells also showed no significant difference in proliferation compared with control plasmid (Fig. 4B)

## 4 Discussion

Driven by the clinical demands for sensitive, reliable, and noninvasive methods to detect primary and recurrent bladder tumors, urologists have made decades-long efforts to discover new markers. However, most of the markers that have been studied in depth are less specific than cytology. By the construction of a urinary bladder cancer cDNA library, we found ten bladder cancer tumor antigens including LBHD1.

Though LBHD1 has a limited effect in human development and human disease itself, LBH, the conserved domain of LBHD1, has been reported to be associated with congenital heart disease [17, 18, 19], rheumatoid arthritis [20], angiogenesis, endochondral bone formation [21], nasopharyngeal carcinoma[22],

and human breast cancers [23, 24]. Moreover, LBH could act as a transcriptional activator in the MAPK signaling pathway [19] and could mediate cellular functions and induce NPC cell cycle arrest at the G1/S transition [22]. Experiments have shown that LBH-homologous LBH transcripts are present in the ectoderm of limb bud. Before the appearance overt morphological signs of limb bud outgrowth, the LBH domain-specific expression pattern indicates that LBH functions in synergy with other genes known to be required for heart and limb development [18]. Interestingly, analyzing the clinical manifestations of LBHD1 gene mutations in human patients in DECIPHER (Database of Chromosomal Imbalance and Phenotype in Humans using Ensembl Resources), we found that partial deletion patients exhibited abnormalities of the foot and abnormalities of the hand.

LBHD1 was identified as a potential tumor antigen which is predominantly expressed in human bladder cancer tissue and localizes predominantly in the cytoplasm by immunohistochemical analysis in our previous research [12]. The role of LBHD1 in bladder cancer development has not been as well characterized. In the present study, we found that LBHD1 is differentially expressed in five different bladder cancer cell lines. LBHD1 showed the lowest expression in the 253J cell line, which is the only noninvasive cell line among the five bladder cancer lines. The biological effects of LBHD1 on bladder cancer cell lines were studied using siRNA gene knockout technology and plasmid overexpression technology. LBHD1 siRNA transfected BLZ-211 cells showed inhibited expression of LBHD1 at the mRNA and protein level, and the downregulation of LBHD1 expression inhibited the migration and invasion of BLZ-211 cells. Similarly, LBHD1 was significantly overexpressed after the LBHD1 plasmid pc-LB was transfected into 253J cells, which promotes cancer cell migration and invasion. Thus, it can be concluded that cell migration and invasion of the BLZ-211 and 253J cell lines are positively correlated with the expression of LBHD1. However, neither downregulation nor overexpression of LBHD1 has detectable influence on cell proliferation. To the best of our knowledge, the data from this study for the first time demonstrates that LBHD1 plays a role in the migration and invasion of human bladder cancer cells.

Collectively, these findings suggest that LBHD1 may be a promising molecular marker for bladder cancer and provide a basis for further analysis of LBHD1 function in bladder cancer migration and invasion. But the mechanism of LBHD1 involvement in bladder cancer is still unclear. Moreover, the levels of LBHD1 expression in surgical specimens and their associations with tumor grade/stage as well as patient outcomes need further study. This study was designed using *in vitro* cell assays and defect immunochemistry assay, so further studies in bladder cancer animal models and surgical specimens are required to validate the findings and hypotheses of this study.
